# Cervical cancer prevention in countries with the highest HIV prevalence: a review of policies

**DOI:** 10.1186/s12889-022-13827-0

**Published:** 2022-08-10

**Authors:** Serra Lem Asangbeh-Kerman, Maša Davidović, Katayoun Taghavi, James Kachingwe, Kereng Molly Rammipi, Laura Muzingwani, Magaret Pascoe, Marielle Jousse, Masangu Mulongo, Mulindi Mwanahamuntu, Neo Tapela, Oluwasanmi Akintade, Partha Basu, Xolisile Dlamini, Julia Bohlius

**Affiliations:** 1 Swiss Tropical and Public Health Institute, Allschwil, Switzerland; 2 University of Basel, Basel, Switzerland; 3 Graduate School for Cellular and Biomedical Sciences, University of Bern, Bern, Switzerland; 4 Graduate School for Health Sciences, University of Bern, Bern, Switzerland; 5 Institute of Social and Preventive Medicine (ISPM), University of Bern, Bern, Switzerland; 6 Malawi Ministry of Health, Lilongwe, Malawi; 7 Principal Health Officer, Ministry of Health, Gaborone, Botswana; 8 International Training and Education Center for Health (I-TECH) Namibia, Windhoek, Namibia; 9 Newlands Clinic, Harare, Zimbabwe; 10 SolidarMed, Chiure, Mozambique; 11 Clinical HIV Research Unit, Wits Health Consortium, Women’s Cancer Research Department, Johannesburg, South Africa; 12 Department of Obstetrics and Gynecology, Women and Newborn Hospital, Lusaka, Zambia; 13 Botswana-Harvard AIDS Institute Partnership, Gaborone, Botswana; 14 Division of Global Health Equity, Brigham and Women’s Hospital, Boston, Massachusetts USA; 15 Elizabeth Glaser Paediatric AIDS Foundation, Maseru, Lesotho; 16 Early Detection, Prevention and Infections Branch, International Agency for Research On Cancer, Lyon, France; 17 Ministry of Health, Mbabane, Eswatini

**Keywords:** Cervical cancer, WLHIV, National policies, Prevention and control, Sub Saharan Africa

## Abstract

**Introduction:**

Cervical cancer (CC) is the leading cause of cancer-related death among women in sub-Saharan Africa. It occurs most frequently in women living with HIV (WLHIV) and is classified as an AIDS-defining illness. Recent World Health Organisation (WHO) recommendations provide guidance for CC prevention policies, with specifications for WLHIV. We systematically reviewed policies for CC prevention and control in sub-Saharan countries with the highest HIV prevalence.

**Methods:**

We included countries with an HIV prevalence ≥ 10% in 2018 and policies published between January 1^st^ 2010 and March 31^st^ 2022. We searched Medline via PubMed, the international cancer control partnership website and national governmental websites of included countries for relevant policy documents. The online document search was supplemented with expert consultation for each included country. We synthesised aspects defined in policies for HPV vaccination, sex education, condom use, tobacco control, male circumcision,cervical screening, diagnosis and treatment of cervical pre-cancerous lesions and cancer, monitoring mechanisms and cost of services to women while highlighting specificities for WLHIV.

**Results:**

We reviewed 33 policy documents from nine countries. All included countries had policies on CC prevention and control either as a standalone policy (77.8%), or as part of a cancer or non-communicable diseases policy (22.2%) or both (66.7%). Aspects of HPV vaccination were reported in 7 (77.8%) of the 9 countries. All countries (100%) planned to develop or review Information, Education and Communication (IEC) materials for CC prevention including condom use and tobacco control. Age at screening commencement and screening intervals for WLHIV varied across countries. The most common recommended screening and treatment methods were visual inspection with acetic acid (VIA) (88.9%), Pap smear (77.8%); cryotherapy (100%) and loop electrosurgical procedure (LEEP) (88.9%) respectively. Global indicators disaggregated by HIV status for monitoring CC programs were rarely reported. CC prevention and care policies included service costs at various stages in three countries (33.3%).

**Conclusion:**

Considerable progress has been made in policy development for CC prevention and control in sub Saharan Africa. However, in countries with a high HIV burden, there is need to tailor these policies to respond to the specific needs of WLHIV. Countries may consider updating policies using the recent WHO guidelines for CC prevention, while adapting them to context realities.

**Supplementary Information:**

The online version contains supplementary material available at 10.1186/s12889-022-13827-0.

## Introduction

Women living with HIV (WLHIV) are at higher risk of developing cervical cancer (CC) compared with HIV negative women [1]. Women with HIV are at higher risk for persistent Human Papillomavirus (HPV) infection, lower chances of clearing infection, faster progression from infection to CC, lower regression of cervical precancerous lesions, and higher recurrence following treatment, compared with HIV negative women [2]. This double HIV-CC burden exacerbates the disparities in CC between High Income Countries (HICs) and Low and Middle Income Countries (LMICs) [3]. In sub-Saharan Africa (SSA), morbidity and mortality rates from both HIV and CC are among the highest globally [1]. In 2018, 38 of the 50 countries with the highest-ranking Population Attributable Fractions (PAF) for CC and HIV were in the African region. The PAF in SSA was 21·0% (15·6–26·8) compared to less than 2% in all other regions globally. The top nine countries with the highest PAFs were in Southern Africa ((PAF: 53·2% (49·1–56·8)) and are included in this review [1, 4]. Effective prevention programmes in HICs have significantly reduced the incidence of CC. However, CC screening in LMICs remains undermined by competing health priorities, resource challenges and lack of monitoring of existing programmes [5, 6].

In November 2020, the World Health Organization (WHO) launched the global strategy for the elimination of CC as a public health problem, defining the 90–70-90 targets [7]. To eliminate CC within a century, 90% of girls should be vaccinated by age 15, 70% of women should be screened with a high precision test by 35 and 45 years of age, and 90% of women with precancerous lesions and invasive CCshould receive treatment and care at national level by 2030. In 2021, the WHO updated guidelines for screening and treatment of cervical precancerous lesions highlighting specific recommendations for WLHIV, including age of first screening for WLHIV [8]. Importantly, the WHO highlights the need for quality control of screening services nationally and globally, including the collection of data to measure standardised process, performance, and impact indicators.

National policies to eliminate CC provide the foundation for the implementation and sustainability of CC screening programmes and demonstrate governments’ commitment to CC prevention and control. In countries with a high HIV burden, this is especially important. Data on country-specific recommendations for CC prevention for WLHIV is rare. We reviewed policies and recommendations for CC prevention and control in SSA countries with the highest HIV prevalence with special focus on the indicators and standards used to monitor the programmes.

## Methods

We conducted a systematic review of national policies, plans, guidelines and strategies for CC prevention and control (simply referred to here as “policies”) in SSA countries with the highest HIV prevalence according to the Preferred Reporting Items for Systematic Reviews and Meta-Analyses (PRISMA) guidelines [9].

### Eligibility criteria for countries and documents

We included countries with an HIV prevalence of 10% and above in 2018 [10]. The prevalence threshold was an arbitrary cut-off for the study. For eligible countries, we identified relevant documents, including national policies, plans, strategies and guidelines for non-communicable diseases (NCDs), cancer, and CC prevention and control without language restrictions published between January 2010 and May 2019. We updated our search in April 2022 to include all policy documents published and unpublished after May 2019 until March 2022. The choice of January 2010 was informed by the oldest policy document in use at the time of the study in Lesotho, which was dated 2011. Documents that did not contain any information on CC prevention were excluded.

### Information sources and searches

We identified documents through a systematic online search and expert consultations. Two researchers systematically searched Medline via PubMed, the web portal of the International Cancer Control Partnership (ICCP), and national (health ministry) websites for included countries between January 2010 and March 2022.

The search terms for PubMed constituted a combination of Medical Subject Heading (MeSH) terms and key words including but not limited to: Human papillomavirus vaccination, cervical cancer screening, cervical cancer prevention, non-communicable diseases control policy OR plan OR strategy OR guideline AND country name. Our search strategy on PubMed was:

(Human Papillomavirus vaccination OR cervical cancer screening OR cervical cancer prevention OR cancer prevention OR non-communicable diseases prevention OR cervical cancer prevention OR cancer prevention OR non communicable diseases prevention) AND (policy or strategy or strategic plan or guidelines or report or directives) AND (Botswana OR Eswatini OR Lesotho OR Malawi OR Mozambique OR Namibia OR South Africa OR Zambia OR Zimbabwe) restricted between January 2010 and May 2019 and updated the search until March 2022. Search terms for health ministry websites included the country name, cervical cancer prevention, cancer prevention and non-communicable diseases control policy, plan, strategy, or guideline. For the ICCP portal, we identified cervical cancer, cancer and NCDs control plans, policies and strategies as well as WHO country profile reports. In addition, we contacted CC prevention and control experts from each included country and asked for any additional relevant documents. Experts from six countries were identified through sites visits conducted by SA within the context of the present study and other studies. Experts from the other three countries not visited (Eswatini, Botswana and Namibia) were recommended by a CC expert in South Africa. We identified global indicators from the Improving Data for Decision-making: a Toolkit for Cervical Cancer Prevention and Control Programmes [11] and the Comprehensive Cervical Cancer Control: a guide to essential practice [12].

### Data charting and management

A data extraction sheet was developed by four researchers (SA, MD, KT and JB), reviewed and approved by the country experts. Data items extracted included policies and protocols related to primary, secondary and tertiary prevention, monitoring and evaluation mechanisms, and service costs to women. The full list of extracted data items is attached in the Additional file 5. We reported indicators for monitoring with a focus on global indicators for CC prevention and control as well as specifications for WLHIV. We also reported corresponding targets, and where available, benchmarks for global indicators. Data was extracted by one reviewer (SA) onto a piloted extraction sheet. All extracted data was cross-checked by a second reviewer (MD). All discrepancies were discussed and resolved.

Reports that were not available in English (documents from Mozambique were in Portuguese) were translated using a translation software, https://www.deepl.com/en/translator and a country expert validated all extracted information. In addition, we consulted one CC prevention expert from each included country for other information pertaining to their CC prevention programmes. We sent two short questionnaires to the experts. The first was a six-item questionnaire extracted from the WHO toolkit for CC prevention and control programmes [11] (Additional file 6). Questions focused on the existence and basic content of policies, plans, and guidelines relevant to CC prevention and control. The second was the WHO 11-item checklist for a comprehensive CC prevention and control program (Additional file 7). This checklist included items on the availability of guidelines for CC prevention specific to WLHIV, availability of financial and technical resources to implement policies, communication strategies to educate the community and advocate for support of national policies, availability of a training plan as well as supervisory mechanisms for quality control and assurance of the programme.

### Data synthesis

We summarised results under five main subheadings: HPV vaccination, sex education, condom use, voluntary medical male circumcision (VMMC) and tobacco control (primary prevention); screening and treatment for cervical pre-cancer lesions (secondary prevention), cervical cancer treatment (tertiary prevention), monitoring and surveillance mechanisms, and costs of services.

The protocol of this review (initially conceived as a scoping review) is registered as a preprint on the African Open Access Portal [13].

### Changes to the protocol

We revised our study design from a scoping review to a systematic review. Consequently, we used the PRISMA guidelines for systematic reviews for reporting and not the PRISMA guidelines for scoping reviews (PRISMA-ScR) as defined in the protocol. Contrary to the exclusion criteria stated in the protocol, (“we will exclude general cancer control plans where a recent standalone CC prevention and control document is available”), we included other cancer, NCD and national plans that contained some information on CC prevention even where there was a standalone policy. Additionally, we contacted country experts for CC prevention policies and related documents. We did not extract definitions of CC indicators as stated in the protocol. We revised our objective and extracted more detailed information on primary, secondary and tertiary prevention of CC as defined by included countries.

## Results

**Fig. 1:**
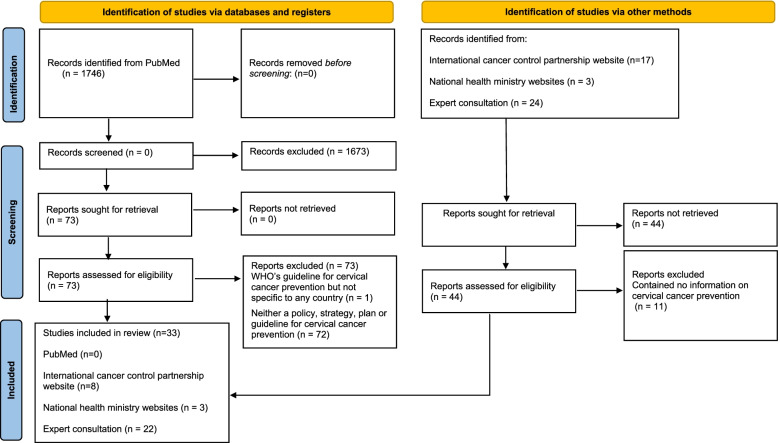
PRISMA flow chart for study selection

We identified nine countries in SSA with HIV prevalence ≥ 10% in 2018: Botswana, Eswatini, Lesotho, Malawi, Mozambique, Namibia, South Africa, Zambia, and Zimbabwe. We identified and reviewed 33 policy documents (Additional file 9). A PRISMA flowchart summarising the document selection process is presented in Fig. 1.

### HPV vaccination, sex education, condom use, voluntary medical male circumcision and tobacco control (primary prevention)

Seven countries reported some aspects of HPV vaccination in their policy documents. Girls between 9–13 years old was the target population for vaccination reported in three of the six countries that defined this item in their policy documents [14, 15]. Reports did not include boys as a target population for vaccination. The school-based vaccination strategy was reported by over a third of the countries (Botswana, Malawi, Namibia and South Africa) [16–21]. Also, two countries had integrated HPV vaccination in the national vaccination programme (Botswana and South Africa) and one was conducting a demonstration programme (Zimbabwe) [22, 23]. Malawi and Zambia were planning to introduce HPV vaccination in the national immunization programme. Only Namibia and Malawi included specifications for HPV vaccination for girls living with HIV in the reviewed documents (Table 1). A three-dose schedule for HPV vaccination was recommended over a 2-dose schedule for the general population of girls [21, 24]. All countries highlighted the need to develop/revise IEC materials for CC prevention. Sex education and warnings about tobacco use were recommended by all countries [15, 16, 22, 24–30]. Condom use was recommended by all countries for the prevention of sexually transmitted infections including HPV [16, 21, 24, 27–29, 31, 32]. In Lesotho and Namibia, we found specific recommendations for condom use.

**Table 1:** Human Papillomavirus (HPV) Vaccination

Country	Target population	Target age (years)	Vaccination strategy	Specifications for girls living with HIV	Integrated in national HPV immunization programme
**Botswana**	Girls	11–13	School-based	NR	Implemented
**Eswatini**^a^	NR	NR	NR	NR	NR
**Lesotho**^a^	NR	9–13	NR	NR	NR
**Malawi**^a^	Girls	9–14 10 (out-of-school)	School and health facility-based	3-dose vaccination schedule	Recommended
**Mozambique**	NR	NR	NR	NR	NR
**Namibia**	Girls	9–14	School-based	3-dose vaccination schedule	NR
**South Africa**	Girls	9–12	School-based	NR	Implemented
**Zambia**	Girls	9–13	NR	NR	Recommended
**Zimbabwe**	Girls	10^b^-14 in school and 10 out of school	NR	NR	Implemented (during demonstration programme)

*NR* is Not Reported

^a^Vaccination not available in these countries (expert response)

^b^Estimated age (reported in policy document as grade 5)

within two weeks after pre-cancer treatment [24, 27]. The promotion of VMMC was highlighted in policy documents in Malawi, South Africa, Namibia and Botswana [20, 28, 33–35] (Additional file 1).

### Screening for and treatment of cervical pre-cancer lesions (secondary prevention)

Most countries recommended cervical screening for WLHIV. The recommended age to start cervical screening for WLHIV varied across countries and differed from the general population in all countries. Zimbabwe recommended starting screening at HIV diagnosis irrespective of age; Namibia recommended starting at 20 years; Lesotho, Malawi and Zambia recommended starting at age 25 years; while Mozambique, and South Africa recommended screening for all ages irrespective of HIV diagnosis [24, 27, 29, 31, 32, 35–38]. There was no information on target age for screening in the general population of women or WLHIV in policy documents in Eswatini. Visual Inspection with Acetic acid (VIA) and Pap smear were the most commonly recommended tests for cervical screening and both were reported in all countries except for Mozambique and Zambia (only VIA reported)[39, 40]. However, the expert in Mozambique we consulted reported that visual inspection with Lugol’s iodine (VILI), Pap and HPV DNA testing are used (Table 2). HPV DNA testing was also recommended in policies in Botswana, Lesotho, Malawi, Namibia, and South Africa. Eight countries reported having histopathology as part of diagnostic services (Table 2). Lesotho did not report any pathology services. Treatment, by cryotherapy or LEEP, was reported in all countries. Eswatini also reported total abdominal hysterectomy for the treatment of cervical precancerous lesions [41]. Cervical screening services were integrated into HIV clinics in Malawi, Namibia, South Africa, Zambia, and Zimbabwe. Integration of cervical screening into HIV clinics and existing services was recommended in Mozambique and Eswatini, respectively [26, 29] (Table 2). The recommended cervical screening interval for WLHIV who screened negative varied between one year (Zimbabwe), two years (Malawi, Lesotho), and three years (Namibia, South Africa, and Zambia). Three countries did not report on this data item. This interval was wider for HIV negative women and varied from three years (Lesotho, Malawi, and Zimbabwe), five years (Namibia, Zambia and Lesotho (for women aged 50 years and older) to ten years in South Africa. Post-treatment follow-up at one year for WLHIV was recommended by Lesotho Malawi, Namibia, South Africa, Zambia, and Zimbabwe. In Malawi and Namibia, routine screening would resume after three negative annual screening while in South Africa, annual screening continued until the woman is lesion free before routine screening was resumed (Table 3).

**Table 2:** Cervical screening, diagnosis and treatment of precancerous lesions

Country	Target age group [years]	Specifications for WLHIV	Entry point	Screening method	Available diagnostic procedures	Treatment of precancerous lesions
Botswana	30–49	NR	NR	VIA, Pap smear, HPV DNA	Colposcopy and histopathology	Cryotherapy, LEEP
Eswatini	NR	NR	Integration into existing services recommended	VIA and Pap smear	Histopathology^b^	Cryotherapy, LEEP, TAH
Lesotho	25–49	Start at HIV diagnosis; Freq.—1 year	NR	VIA, VILI, Pap smear, HPV DNA testing	Not available	Cryotherapy, cold coagulation, LEEP
Malawi	25–49	Start age 25 21–24 upon request Freq.—2 years	HIV clinic, SRH	VIA, Pap smear, HPV DNA testing	Histopathology	Cryotherapy, cold coagulation, surgery, LEEP
Mozambique	NR	Screen all ages	Integration of HIV and CC screening services recommended	NR^a^	Histopathology	Cryotherapy, surgery
Namibia	25–50	Start at age 20	HIV clinics, ANC clinics	VIA, Pap smear, HPV DNA testing	Colposcopy and histopathology	Cryotherapy, LEEP, Thermocoagulation
South Africa	30–55	Screen all ages	Family planning, HIV clinic	LBC, Pap smear, VIA, HPV DNA testing	Histopathology	LEEP, Cryotherapy for low-resource settings
Zambia	30–59	Start age 25	HIV clinic, MCH	VIA	Histopathology	Cryotherapy, LEEP
Zimbabwe	30–49	Start at HIV diagnosis; Freq.—1 year	Family planning, maternity, Gynaecological clinics, HIV clinics	VIAC, Pap smear^c^	Histopathology	Cryotherapy, LEEP

*ANC* is Antenatal clinic, *MCH* is Mother and Child Health clinic, *SRH* is Sexual and Reproductive Health unit, *Freq.* is frequency, VIA is Visual inspection with acetic acid, *VILI* is Visual Inspection with Lugol’s Iodine, *LBC* is Liquid based cytology, *LEEP* is Loop electrosurgical excision procedure, *LLETZ* is Large loop excision of the transformation zone, *TAH* is Total abdominal hysterectomy *NR* is Not reported

^a^VILI, Pap, HPV DNA testing (expert report)

^b^Expansion of services ongoing

^c^Pap smear mostly done in private health facilities

**Table 3:** Post screening/treatment follow-up

Recommended rescreening interval				
**Country**	**Screen negative**		**After treatment**	
	**HIV negative**	**HIV positive**	**HIV negative**	**HIV positive**
**Botswana**	NR	NR	NR	NR
**Eswatini**	NR	NR	NR	NR
**Lesotho**	3–5 years	2 years	1 year post treatment then return to routine screening	6 months post treatment then yearly screening thereafter
**Malawi**	3 years	2 years	Yearly for 3 years then return to routine screening	
**Mozambique**	NR	NR	NR	NR
**Namibia**	5 years	3 years	Yearly for 3 consecutive years then return to routine screening	Yearly for 3 consecutive years then return to routine screening
**South Africa**	10 years	3 years	Yearly	Yearly until lesion-free then return to routine screening
**Zambia**	5 years	3 years	NR	Yearly
**Zimbabwe**	3 years	yearly	1 year post treatment	1 year post treatment

*NR* is not reported

### Treatment of invasive cervical cancer (tertiary prevention)

Available treatment services for invasive CC were reported in six countries: surgery (South Africa, Malawi, radiotherapy (Botswana, Mozambique, Namibia, South Africa, Zambia, and Zimbabwe) and chemotherapy (Botswana, Malawi, South Africa and Zambia). Palliative care was reported available in seven countries; Additional file 2). Lesotho reported the need to have treatment services available in-country. Women diagnosed with invasive cancer were referred to South Africa for treatment [27].

### Monitoring and surveillance mechanisms

Data systems were either paper-based (Lesotho, Malawi, Mozambique) or a combination of electronic and paper-based (Botswana, Namibia, South Africa, Zambia). Eswatini reported transitioning to an electronic data system while Zimbabwe did not explicitly state the nature of their data collection system (Table 4). We checked whether the countries report the four global indicators recommended by the WHO for monitoring CC elimination progress: HPV vaccination coverage, screening rate, screening test positivity rate and treatment rate, and whether these indicators were disaggregated by HIV status (Table 5). We also identified and reported corresponding national targets and/or benchmarks for these indicators, when available. All countries defined indicators for monitoring CC prevention and control programmes, and we listed them in (Additional file 8). At least two global indicators were reported in all countries except Eswatini. Only four countries reported these indicators explicitly disaggregated by HIV status. Five other national indicators recommended by Zimbabwe were also disaggregated by HIV status. The number of WLHIV screened for precancerous lesions was reported in Botswana, Malawi and Namibia. VIA test positivity was disaggregated by HIV status in Botswana, Mozambique and Namibia. Treatment rate by HIV status was reported in Botswana and Namibia. Other recommended national indicators disaggregated by HIV status were: percentage of health facilities providing integrated HIV/sexually transmitted infections (STI)/CC screening, number of staff trained in integrated CC/breast/HIV/STI service per facility, percentage of facilities with HIV/STI and cancer integrated services, percentage of clients accessing integrated HIV/STI and cancer services, number of staff trained in integrated cancer/HIV/STI early detection and management services. Targets were defined for the general population of women, with no specificities for WLHIV. There were no details in reviewed documents describing how these targets were arrived at. In Malawi however, their updated guideline included a revised target for screening coverage from 80 to 70% “to reflect WHO targets” [21]. Targets for HPV vaccination ranged from 80% (Zambia and Zimbabwe) to 100% (South Africa). Targets for screening coverage ranged from 65% (South Africa) to 80% (Botswana, Malawi, Mozambique, Namibia). Zambia defined a benchmark of 5–10% test positivity rate for screened women. Treatment coverage targets ranged between 80% (Botswana, Zambia for treatment of cervical precancerous lesions by cryotherapy and treatment of CC) and 95% (Zambia for LEEP). Cancer registries were present in all countries but Lesotho [15, 21, 28–30, 33, 37, 42]. CC data registration was done in all eight countries although with some limitations. Most registries were not population-based (Malawi, Eswatini, Zambia, and/or located only in major cities (Zimbabwe, Mozambique). Malawi highlighted the need to enhance linkages between the CC prevention programme and the cancer registry while Eswatini recommended strengthening cancer data registration. Zambia sought to strengthen cancer registration by making cancer a notifiable disease and expanding from hospital-based registries to population-based.

**Table 4:** Monitoring and surveillance systems

Country	Referral system	Data system	Cancer register
Botswana	Available	Electronic and paper-based	Available
Eswatini	Lack of referral system	Transition to electronic data collection ongoing	Available
Lesotho	System available with referral logbook	Paper based	NR^c^
Malawi	Structured system with algorithm	Paper based	Available
Mozambique	NR^b^	Paper based	Available
Namibia	Available	Electronic and paper based	Available
South Africa	Structured system	Electronic and paper based	Available
Zambia	Design of referral systems recommended	Electronic and paper based	Available
Zimbabwe	Not fully functional	NR^a^	Available

*NR* is not reported

Experts’ reports:

^a^Available but needs strengthening;

^b^Not available;

^c^Not available

**Table 5:** Indicators and targets for cervical cancer prevention and control

Country	WHO global indicators					Country targets					
	**Vaccination rate**	**Screening rate**	**Screening test positivity rate**		**Treatment rate**	**Vaccination coverage**	**Timeline**	**Screening coverage**	**Timeline**	**Treatment coverage**	**Timeline**
**Botswana**	✓	✓^a^	✓^a^		✓^a^	98%	2025	80%	2022	80%	2022
**Eswatini**	☓	☓	☓		☓	☓	☓	☓	☓	☓	☓
**Lesotho**	✓	✓	✓		✓	90%	☓	☓	☓	☓	☓
**Malawi**	✓	✓^a^	✓		✓	95%	2026	70%	2026	85%	2026
**Mozambique**	☓	✓	✓^a^		☓	☓	☓	80%	☓	☓	☓
**Namibia**	✓	✓^a^	✓^a^		✓^a^	95%	☓	80%	2025	☓	☓
**South Africa**	✓	✓	✓		✓	100%	✓	65%	☓	☓	☓
**Zambia**	✓	✓	✓		✓	80%	☓	^b^5-10%	Monthly	80%:Cryotherapy: 95%:LEEP: > 80%:ICC treatment	80%
**Zimbabwe**	✓	✓	☓		☓	80%	2018	☓	☓	☓	2020
	**Other indicators disaggregated by HIV status**										
**Zimbabwe**	Percentage of health facilities providing integrated HIV/STI/CC screening			Number of staff trained in integrated CC/breast/HIV/STI service per facility			Percentage of facilities with HIV/STI and cancer integrated services			Percentage of clients accessing integrated HIV/STI and cancer services	
				Number of staff trained in integrated cancer/HIV/STI early detection and management services							

^a^Disaggregation of data point by HIV status

^b^Test positivity benchmark for new women screened; WHO’s timeline for 70–90-70 targets is 2030

### Costs of services to women

Three countries reported the costs of HPV vaccination in their policy documents. This service was free of charge in Malawi and in government facilities in Lesotho. In South Africa, HPV vaccination was free only in schools. Cervical screening services were free in Lesotho, Malawi, South Africa, Mozambique and Namibia (expert report) and free for vulnerable groups in Botswana. Diagnostic services were free for vulnerable groups in Botswana and in public facilities in South Africa. These services were paid for by women in Zimbabwe and were expensive (expert report, Additional file 3). Treatment for cervical precancerous lesions was free in Malawi and South Africa, for vulnerable groups in Botswana, and in some health facilities in Zimbabwe. Treatment for invasive CC was free in public health facilities in South Africa while these services were unaffordable in Zimbabwe (Additional file 3). Other countries did not report on costs of treatment.

## Discussion

In this review, we evaluated and summarised policies and recommendations for CC prevention and control in the nine SSA countries with the highest HIV prevalence. All countries reviewed had cancer control policies containing aspects of CC prevention. There was considerable variation in the surveyed countries’ recommendations for CC prevention among WLHIV. The most common reported age group for HPV vaccination was 9–13 years, with specific considerations for girls/women living with HIV recommended in Namibia. The school-based strategy was the most common vaccination strategy. Integration of HPV vaccination into national immunisation programmes was less common. Sex education, promotion of condom use for sexually active individuals and warnings against tobacco use was recommended in all countries, while VMMC was recommended in four countries. Age to start cervical screening varied across policies. Dominant reported methods for cervical screening, pre-cancer diagnosis, and treatment were VIA, Pap smear testing, histopathology, cryotherapy, and LEEP. Cervical screening was mostly integrated into gynaecological units, HIV clinics, family planning units, and mother and child health units and was not always free of charge. Invasive CC treatment recommendations for WLHIV were not common in the policies reviewed. Data systems for monitoring were widely available in the countries studied. There was a general lack of HIV-disaggregated indicators for monitoring.

### Comparison with WHO guidelines and current evidence

The most common age group reported for HPV vaccination in policy documents lies within WHO’s recommended age group for HPV vaccination, i.e. 9–14 years [8]. However, the WHO’s suggestion that HPV vaccination should be implemented within national immunisation programmes was not commonly practised by the countries we surveyed. A recent report on HPV vaccination programmes in LMICs showed that five of the countries included in this review (Malawi, Zambia, Zimbabwe, Botswana, and South Africa) had introduced HPV vaccination, partially or nationwide [43] which corresponds to information extracted from their policy documents. The Vaccines in National Immunisation Programmes report of 2019, indicated that four of these countries (Botswana, South Africa, Zambia, and Zimbabwe) had integrated HPV vaccination in their national immunisation programme [44], which also corroborated their policy documents. The exception to this trend was Zambia, which reported this aspect as ‘recommended’ [15]. These disparities in reports between country policy documents and other published reports may be explained by changes in practice not yet captured in these documents. We found specific recommendations for HPV vaccination for girls living with HIV only in Namibia and Malawi. Recent evidence suggests that a one-dose vaccination schedule for girls in the general population is effective against persistent HPV infection [45]. For girls living with HIV, whether the number of doses can be reduced to two remains unclear. However, WHO suggests to continue with three doses [46]. Although HPV vaccination has been shown to be beneficial in reducing incidence rates of all HPV-related disease among the female and male populations [47, 48], vaccination for boys may not be the best investment in limited health care resources contexts [49, 50]. Limited financial resources was highlighted in policy documents in Malawi, Mozambique, Namibia and South Africa as a barrier to extension of vaccination services to boys [16, 21, 28, 29]. The school-based vaccination strategy has also been shown to achieve higher coverage compared to other strategies [43]. Consistent condom use and VMMC offer some protection against HPV transmission [51–55]. There is also evidence that consistent condom use improves the chances of regression of cervical intraepithelial neoplasia [56]. Both primary prevention methods are recommended by WHO for the prevention of HIV and other STIs [57, 58]. Smoking negatively affects HPV disease progression [59]. Tobacco use reduction is one of WHO’s “best buys” for primary prevention of NCDs including CC [60]. All countries aligned their tobacco control policy with WHO’s Framework Convention for Tobacco Control (FCTC), recommending price and tax measures to reduce demand for tobacco, bans on advertising and sponsorship as well as education, and public awareness amongst other strategies [61]. The WHO recommends that WLHIV are screened with the HPV DNA test rather than VIA or cytology [8]. While HPV DNA testing was available in five countries, VIA and cytology were the most common tests used. These results are consistent with those reported in a review of policies in East African countries where the CC burden is also high [62]. Lesotho, Malawi and Zambia advise commencing screening from 25 years for WLHIV, which aligns with WHO’s updated recommendations [8]. There is also a need to balance cost-effectiveness with the socio-cultural specificities of a given country. For example, Mozambique and South Africa advise screening WLHIV of all ages which may not be cost-effective but may be advisable given the tendency for early sexual debut [63, 64]. Treatment of CC in WLHIV also requires some considerations as chemo radiation in immunocompromised women (including WLHIV) may pose some challenges [65]. The increased risk of recurrent infection after treatment in WLHIV also requires shorter follow-up intervals compared to women in the general population [8]. The WHO provides guidance for disaggregating monitoring indicators by HIV status, to identify gaps for this vulnerable population and corrective measures for prevention and control programmes [11]. Monitoring cervical screening in LMICs is challenging. One of the major barriers is the lack of formal screening registries used in high-income countries such as England, Australia, and New Zealand [66]. Electronic systems for data collection were less common in the countries studied. These systems reduce cost and time for data collection, management, and patient monitoring [67]. In several SSA countries, the predominance of paper-based monitoring systems limits the collection of comprehensive CC data including indicators disaggregated by HIV status. Also, national CC prevention and control activities are usually dispersed across different bodies or units (like NCDs and sexual and reproductive health) without centralised data systems, making monitoring even more complex and challenging [6]. For example, a recent mid-term review of Zimbabwe’s CC prevention and control strategy revealed that there was no data on the proportion of women who ever tested for CC, which existed nationally, and data on investigations were not routinely collected at the national level [68].

The WHO also recommends integrating HIV and CC services. This will allow service providers to increase their skills and knowledge, improving care for a high-risk population and at the same time reducing stigma, and improving cost-effective use of resources [69, 70].

Funding for CC prevention is limited which implies that CC prevention services are not always free [66], creating a barrier to access. This could be addressed through strengthening partnerships with organisations such as UNAIDS, the International Atomic Energy Agency and the Go Further partnership to end AIDS.

### Strengths and weaknesses

Our analysis was strengthened by the exhaustive search and data collection achieved. We supplemented online searches with policy documents, published and unpublished, and direct communication with country experts. We reported data items and indicators that present the full continuum of CC prevention and care.. We only reported indicators disaggregated by HIV status as seen in policy documents. However, in practice, some programmes, especially those integrated in HIV care, probably collect CC and HIV data with the ability to report indicators for WLHIV. Additionally, our focus on global indicators for monitoring progress toward CC elimination in this analysis does not account for indicators defined for subnational and national monitoring of CC prevention and control programmes. Furthermore, experts’ responses may only reflect the programmes they work for and may not be representative of national practices. Some of the information collected through this systematic review process may have changed.

## Conclusion

Many SSA countries are making strides with policy development and implementation for CC prevention and control. However, current national policies for CC prevention and control in SSA countries with a high HIV burden could be better tailored to the specific needs of women living with HIV. This review highlights gaps in CC prevention and control recommendations for WLHIV in nine SSA countries. To better adapt to the needs of WLHIV, these countries need to update their policies using the WHO’s recent guidelines, while adapting them to context realities. Well defined indicators, appropriately disaggregated by HIV and monitoring all steps of CC continuum of care will further address gaps in CC control among WLHIV.

## Supplementary Information

**Additional file 1.** Other Primary prevention strategies (Sex education, condom use, circumcision and tobacco control)

**Additional file 2.** Treatment of invasive cervical cancer and palliative care

**Additional file 3.** Cost of services for clients

**Additional file 4.** Other responses from country experts (some questions from appendices 2 and 3)

**Additional file 5.** Data extraction sheet

**Additional file 6.** Extract from WHO’s CC prevention and control toolkit for cervical cancer prevention and control programmes [11]

**Additional file 7. **WHO checklist for a comprehensive cervical cancer prevention and control programme [12]

**Additional file 8.** List of indicators and targets extracted from included policy documents

**Additional file 9.** List of documents reviewed

**Additional file 10.** Age standardised cervical cancer incidence and mortality rates for included countries

## Data Availability

The supplementary files contain most of the data generated and analysed during the current study. Other details on data for this study are available from the corresponding author on reasonable request.

## Abbreviations

AIDS: Acquired Immunodeficiency Syndrome
CC: Cervical Cancer
CIN: Cervical Intraepithelial Neoplasia
DNA: Deoxyribonucleic acid
HIV: Human Immunodeficiency Virus
HGSIL: High Grade Squamous Intraepithelial Lesion
HPV: Human Papillomavirus
ICCP: International Cancer Control Partnership
LBC: Liquid-Based Cytology
LEEP: Loop Electrosurgical Excision Procedure
LMICs: Low and Middle Income Countries
NCDs: Non Communicable Diseases
Pap test: Papanicolaou test
PRISMA: Preferred Reporting Items for Systematic Reviews and Meta-Analyses
SSA: Sub-Saharan Africa
STI: Sexually Transmitted Infections
UNAIDS: The Joint United Nations Programme on HIV/AIDS
VIA: Visual Inspection with Acetic Acid
VIAC: Visual Inspection with Acetic acid and Cervicography
VILI: Visual Inspection with Lugol’s Iodine
WHO: World Health Organization
WLHIV: Women Living with HIV
