# Supplementary material for: Cervical cancer prevention in countries with the highest HIV prevalence: a review of policies
Source: BMC Public Health. 2022 Aug 10;22:1530. doi: 10.1186/s12889-022-13827-0 (PMC9367081; doi:10.1186/s12889-022-13827-0)
Supplement: Supplementary file 1 — Additional file 1. Other Primary prevention strategies (Sex education, condom use, circumcision and tobacco control) [file 12889_2022_13827_MOESM1_ESM.docx]

**Additional file 1: Other Primary prevention strategies (Sex education, condom use, circumcision and tobacco control)**

| **Country** | **Sex education (development of IEC material)** | **Condom Use** | **Voluntary Male Medical Circumcision** | **Warnings about tobacco use** |
| --- | --- | --- | --- | --- |
| **Botswana** | √ | √ | √ | √ |
| **Eswatini^£^** | √ | √ | χ | √ |
| **Lesotho^£^** | √ | √ | χ | √ |
| **Malawi^£^** | √ | √ | √ | √ |
| **Mozambique** | √ | √ | χ | √ |
| **Namibia** | √ | √ | √ | √ |
| **South Africa** | √ | √ | √ | √ |
| **Zambia** | √ | √ | χ | √ |
| **Zimbabwe** | √ | √ | χ | √ |

√ is recommended or present
