# Supplementary material for: Cervical cancer prevention in countries with the highest HIV prevalence: a review of policies
Source: BMC Public Health. 2022 Aug 10;22:1530. doi: 10.1186/s12889-022-13827-0 (PMC9367081; doi:10.1186/s12889-022-13827-0)
Supplement: Supplementary file 2 — Additional file 2. Treatment of invasive cervical cancer and palliative care [file 12889_2022_13827_MOESM2_ESM.docx]

**Additional file 2: Treatment of invasive cervical cancer and palliative care**

| **Country** | **Treatment of invasive cancer** | **Palliative care** |
| --- | --- | --- |
| **Botswana** | Radiotherapy | Available |
| **Eswatini** | NR | Available |
| **Lesotho** | Not available  (Treatment of invasive cancer done in South Africa) | Available, centralised |
| **Malawi** | Surgery, chemotherapy | Available, centralised |
| **Mozambique** | NR | NR |
| **Namibia** | Surgery, chemotherapy, radiotherapy | NR |
| **South Africa** | Surgery, chemotherapy, radiotherapy | Available |
| **Zambia** | Chemotherapy, radiotherapy | Available |
| **Zimbabwe** | Radiotherapy  (Treatment for invasive cancer is mostly done in private health facilities at high cost) | Available, centralised |
| NR is not reported |  |  |
