# Supplementary material for: Cervical cancer prevention in countries with the highest HIV prevalence: a review of policies
Source: BMC Public Health. 2022 Aug 10;22:1530. doi: 10.1186/s12889-022-13827-0 (PMC9367081; doi:10.1186/s12889-022-13827-0)
Supplement: Supplementary file 3 — Additional file 3. Cost of services for clients [file 12889_2022_13827_MOESM3_ESM.docx]

**Additional file 3: Cost of services for clients**

| **Country** | **HPV vaccination** | **Cervical screening** | **Diagnostic procedures** | **Treatment of cervical pre-cancer** | **Treatment of**  **invasive cancer** |
| --- | --- | --- | --- | --- | --- |
| **Botswana** | NR | Free for vulnerable groups | Free for vulnerable groups | Free for vulnerable groups | NR |
| **Eswatini*** | NR | NR | NR | NR | NR |
| **Lesotho** | Free in government facilities | Free | NR | NR | NR |
| **Malawi** | Free | Free | NR | Free | NR |
| **Mozambique** | NR | NR | NR | NR | NR |
| **Namibia*** | NR | NR | NR | NR | NR |
| **South Africa** | Free in school and about $65 out of school | Free | Free in public facilities | Free | Free in public facilities |
| **Zambia** | NR | NR | NR | NR | NR |
| **Zimbabwe** | NR | Free | ^a^Unaffordable | Treatment is charged in some institutions (particularly LEEP) | ^a^Prohibitive |

*Financial and technical resources are not available to ensure services are available and affordable to women (experts’ report)

^a^As reported by country expert

NR is not reported
