# Supplementary material for: Cervical cancer prevention in countries with the highest HIV prevalence: a review of policies
Source: BMC Public Health. 2022 Aug 10;22:1530. doi: 10.1186/s12889-022-13827-0 (PMC9367081; doi:10.1186/s12889-022-13827-0)
Supplement: Supplementary file 4 — Additional file 4. Other responses from country experts (some questions from appendices 2 and 3) [file 12889_2022_13827_MOESM4_ESM.docx]

**Additional file 4: Other responses from country experts (some questions from appendices 2 and 3)**

**Item/service**

| **Country** | **1** | **2** | **3** | **4** | **5** | **6** | **7** | **8** | **9** | **10** |
| --- | --- | --- | --- | --- | --- | --- | --- | --- | --- | --- |
| **Botswana** | √ | √ | √ | √ | Partially available | √ | Unable to comment | Unable to comment | Needs strengthening | Needs strengthening |
| **Eswatini*** | √ | √ | √ | χ | χ | χ | χ | χ | χ | χ |
| **Lesotho** | √ | χ | χ | √ | √ | √ | χ | √ | √ | χ |
| **Malawi** | √ | √ | √ | √ | √ | √ | √ | √ | √ | √ |
| **Mozambique** | √ | χ | χ | √ | √ | √ | χ | √ | χ | χ |
| **Namibia*** | √ | √ | χ | √ | √ | χ | χ | √ | √ | χ |
| **South Africa** | √ | √ | √ | √ | √ | √ | √ | √ | √ | √ |
| **Zambia** | √ | √ | √ | √ | χ | √ | χ | √ | √ | √ |
| **Zimbabwe** | √ | √ | χ | √ | √ | √ | √ | √ | √ | √ |

1. Single visit approach recommended
2. Clinical practice guidelines for CC screening specific to HIV infected women
3. Guidelines for HIV infected women separate document from clinical practice guidelines
4. Functional multidisciplinary platform to foster partnership and collaboration and set the national agenda
5. National guidelines for health workers for all components of comprehensive cervical cancer prevention and control
6. Financial and technical resources to implement the policy and plan and ensure that services are available and affordable to girls and women
7. Communication strategies to educated the community and advocate for support of national policies
8. A training plan in place as well as supervisory mechanisms for quality control and assurance of the programme
9. A functional referral system that links screening services with the treatment of precancerous lesions and invasive cancer
10. Functioning monitoring systems to track coverage of HPV vaccination, screening and follow-up treatment
