# Supplementary material for: Cervical cancer prevention in countries with the highest HIV prevalence: a review of policies
Source: BMC Public Health. 2022 Aug 10;22:1530. doi: 10.1186/s12889-022-13827-0 (PMC9367081; doi:10.1186/s12889-022-13827-0)
Supplement: Supplementary file 5 — Additional file 5. Data extraction sheet [file 12889_2022_13827_MOESM5_ESM.docx]

**Additional file 5**: Data extraction sheet

| General information | Country |
| --- | --- |
|  | Title of policy document |
|  | Source (e.g. www location) |
|  | Period of validity |
| Human papillomavirus vaccination | Recommended vaccine |
|  | Target population and age |
|  | Vaccination strategy |
|  | Cost for clients |
|  | Integrated in national programme on immunization (Yes/No) |
|  | Indicators for monitoring vaccination programme |
|  | Targets |
| Screening and treatment of cervical precancerous lesions and invasive cancer | Organised or Opportunistic |
|  | Target age group |
|  | Specifications for WLHIV? |
|  | Entry point for screening (family planning, HIV clinic, STI clinic) |
|  | Screening method (s) |
|  | Cost of screening for clients |
|  | Diagnostic capacity (present/absent/rare) |
|  | Cost of diagnosis for clients |
|  | Treatment for precancerous lesions (cryo, cold coagulation, surgery) |
|  | Cost of treatment of pre-cancer for clients |
|  | Treatment for invasive cervical cancer (radio, chemo, surgery, not available) |
|  | Cost of treatment of invasive cervical cancer for clients |
| Follow-up | Follow-up intervals for screen-positive and screen-negative women defined (Yes/No). |
|  | Palliative care (available/not available) |
|  | Cancer registry (present/absent) |
|  | Indicators for monitoring screening, treatment and follow-up |
|  | Targets for screening, treatment and follow-up |
| Availability of data systems | Data entry (electronic/paper-based) |
| Indicators and targets | Indicators and targets for CC prevention will be extracted |
