# Supplementary material for: Cervical cancer prevention in countries with the highest HIV prevalence: a review of policies
Source: BMC Public Health. 2022 Aug 10;22:1530. doi: 10.1186/s12889-022-13827-0 (PMC9367081; doi:10.1186/s12889-022-13827-0)
Supplement: Supplementary file 6 — Additional file 6. Extract from WHO’s CC prevention and control toolkit for cervical cancer prevention and control programmes [11] [file 12889_2022_13827_MOESM6_ESM.docx]

| **Additional file 6**: Extract from WHO’s CC prevention and control toolkit for cervical cancer prevention and control programmes (11) | |
| --- | --- |
| **Question** | **Response options** |
| Is there a national health policy, plan or strategy? Does it include cervical cancer prevention and control? | HPV Vaccination |
|  | Screening PCL treatment |
|  | Invasive Cervical Cancer |
|  | Does not address cervical cancer prevention and control |
| Is there a national policy, plan or strategy for cancer prevention and control? Does it include cervical cancer prevention and control? | HPV Vaccination |
|  | Screening PCL treatment |
|  | Invasive Cervical Cancer |
|  | Does not address cervical cancer prevention and control |
| Is there a policy, plan or strategy specific to cervical cancer (in addition to the national cancer prevention and control policy)? What does it cover? | HPV Vaccination |
|  | Screening PCL treatment |
|  | Invasive Cervical Cancer |
|  | Does not address cervical cancer prevention and control |
| If policies, plans or strategies which address cervical cancer prevention and control exist, what cervical cancer screening method do they recommend? | Cytology/Pap smear |
|  | VIA |
|  | VILI |
|  | HPV DNA test Other (specify): |
|  | No recommendation |
| What method for the treatment of precancerous lesions is recommended by policies, plans or strategies which address cervical cancer? | Cryotherapy |
|  | LEEP |
|  | Conization |
|  | Thermal/cold coagulation |
|  | Other (specify): |
|  | No recommendation |
| Is a Single Visit Approach for cervical cancer screening and precancerous lesion treatment recommended by policies, plans or strategies? | □ Yes □ No |
| Are there standardized national clinical practice guidelines for the following cervical cancer services? | Screening |
|  | □ Yes □ No |
|  | Treatment of precancerous lesions Management of invasive cervical cancer |
|  | □ Yes □ No |
|  | □ Clinical practice guidelines do not exist for cervical cancer services |
| Are there clinical practice guidelines for cervical cancer screening specific to HIV infected women? | □ Yes □ No |
| If Yes, are these guidelines a separate document from the clinical practice guidelines for screening noted above? | □ Yes □ No |
