# Supplementary material for: Cervical cancer prevention in countries with the highest HIV prevalence: a review of policies
Source: BMC Public Health. 2022 Aug 10;22:1530. doi: 10.1186/s12889-022-13827-0 (PMC9367081; doi:10.1186/s12889-022-13827-0)
Supplement: Supplementary file 7 — Additional file 7. WHO checklist for a comprehensive cervical cancer prevention and control programme [12] [file 12889_2022_13827_MOESM7_ESM.docx]

| **N^o^** | **Item** |
| --- | --- |
| 1 | Functional multidisciplinary platform to foster partnership and collaboration and set the national agenda |
| 2 | Comprehensive national policy or plan on cervical cancer prevention and control |
| 3 | National guidelines for health workers for all components of comprehensive cervical cancer prevention and control |
| 4 | Financial and technical resources to implement the policy and plan and ensure that services are available and affordable to girls and women |
| 5 | Communication strategies to educated the community and advocate for support of national policies |
| 6 | A training plan in place as well as supervisory mechanisms for quality control and assurance of the programme |
| 7 | HPV vaccination as a population based strategy to an appropriate cohort in the target age group of 9 and 13 year old girls |
| 8 | Cervical cancer programme to screen and treat every woman between 30 and 49 years old at least once in their lifetime |
| 9 | A functional referral system that links screening services with the treatment of precancerous lesions and invasive cancer |
| 10 | Functioning monitoring systems to track coverage of HPV vaccination, screening and follow-up treatment |
| 11 | Existence of a cancer registry as part of the health information system to monitor cervical cancer incidence and mortality |

**Additional file 7**: WHO checklist for a comprehensive cervical cancer prevention and control programme (12)
