# Supplementary material for: Cervical cancer prevention in countries with the highest HIV prevalence: a review of policies
Source: BMC Public Health. 2022 Aug 10;22:1530. doi: 10.1186/s12889-022-13827-0 (PMC9367081; doi:10.1186/s12889-022-13827-0)
Supplement: Supplementary file 8 — Additional file 8. List of indicators and targets extracted from included policy documents [file 12889_2022_13827_MOESM8_ESM.docx]

**Additional file 8: List of indicators and targets extracted from included policy documents**

| **Country** | **Plan title** | **Indicators** | **Targets** |
| --- | --- | --- | --- |
| Malawi | National cervical cancer control strategy | HPV vaccine coverage rate: Percentage of girls aged 9-13years who have received all the doses of the HPV vaccine in the previous 12-month period | 90% |
|  |  | Screening coverage rate: Percentage of women 25-49 years who have been screened with VIA for the first time with in the previous 12-month period | 80% |
|  |  | Treatment rate for VIA positive women: percentage of VIA- positive women receiving treatment in the previous 12-month period | 90% |
|  |  | Treatment of cancers: percentage of curable cervical cancer patients receiving adequate care | 10% by 2020 |
|  |  | Percentage of women receiving palliative care for advanced cervical cancer | 50% by 2020 |
|  |  | Decreased incidence from invasive cervical cancer |  |
|  |  | Decreased mortality from invasive cervical cancer |  |
|  | Standard Operating Procedures for CC services | Number of clients screened for cervical cancer disaggregated by age |  |
|  |  | Number of clients screened for cervical cancer disaggregated by HIV status |  |
|  |  | Number of clients screened disaggregated by reason for facility visit |  |
|  |  | Number of clients screened disaggregated by screening method |  |
|  |  | Cervical cancer screening results |  |
|  |  | Number of cancer suspects disaggregated by age |  |
|  |  | Total number of clients treated |  |
|  |  | Number of clients treated disaggregated by treatment option |  |
|  |  | Number of clients referred disaggregated by referral reasons |  |
|  |  | Number of clients that received feedback |  |
|  |  | Percentage of facilities providing cancer screening service |  |
|  |  | Number of cervical cancer service providers trained |  |
|  |  | Number of active service providers during the past three months |  |
|  | National cancer prevention and control strategy | Percentage of facilities providing cancer screening, early detection and linkages to care | Increase by 60% the number of facilities providing early detection and treatment services |
|  |  | Percentage of level 3-5 facilities offering basic cancer diagnosis, treatment and palliative care | Expand to 80% the number of level 3-5 facilities offering basic cancer diagnosis, treatment and palliative care by 2022 |
|  |  | Number of facilities with systems in place to meet the requirements for cancer surveillance, research, and strategic information systems | To strengthen cancer surveillance, research and strategic information systems |
|  |  | Number of facilities that are well-equipped with the proper infrastructure, specialists, and technologies for cancer prevention and control | Number of facilities that are well-equipped with the proper infrastructure, specialists, and technologies for cancer prevention and control |
|  |  | Number of improved policies or partnerships established for prevention, treatment, care and rehabilitation of cancer | To establish a high level mechanisms for multi-sectoral coordination and partnership for prevention, treatment, care and rehabilitation of cancer |
|  |  | CC incidence |  |
|  |  | CC mortality |  |
|  | Non-communicable diseases annual programme report | Proportion of CC deaths related to inpatient admissions |  |
|  | National Cervical Cancer Strategic plan | Cervical cancer age-standardised incidence rate | 60/100000 by 2026 (same target for all indicators listed in this plan) |
|  |  | Cervical cancer Age Standardized mortality rate | 40/100000 |
|  |  | Cervical cancer included on government funding budget line items for MOH |  |
|  |  | Number of facilities providing cervical cancer control services | 150 |
|  |  | Number of CSOs actively engaged in advocacy in cervical cancer control activities | 40 |
|  |  | Established and operational social grants for cancer patients to access treatment |  |
|  |  | A costed implementation plan for the cervical cancer control strategy | 1 |
|  |  | Number of strategic plan dissemination meetings conducted (including launch of the strategic plan) |  |
|  |  | Number of people trained per category e.g. CSOs affiliates, peers, youth etc. | 700 |
|  |  | Published IEC materials |  |
|  |  | Number of community representatives engaged in cervical cancer public awareness activities (including traditional leaders, church leaders etc) | 60 |
|  |  | Number of active cervical cancer champion programs in the country | 40 |
|  |  | Number of cervical cancer patients enrolled in the champion programs | 400 |
|  |  | Number of public cervical cancer awareness events conducted | 25 |
|  |  | Number of role models participating in cervical cancer prevention activities | 25 |
|  |  | Number of males attending public cervical cancer awareness events | 3000 |
|  |  | Topics on cervical cancer prevention included in the curricula for primary and secondary schools |  |
|  |  | Number of orientation meeting conducted | 4 |
|  |  | Number of people oriented in effective messaging of cervical cancer prevention and control | 300 |
|  |  | Percentage of eligible adolescent girls who received the HPV vaccine | 95 |
|  |  | Percentage of health facilities offering HPV vaccine | 90 |
|  |  | Percentage of health facilities without stock out of HPV vaccine among those offering HPV vaccine | 90 |
|  |  | Number of adolescents vaccinated through outreach clinics, village clinics or mobile clinics | 200000 |
|  |  | Number of health facilities offering HPV vaccine in out of facility settings | 950 |
|  |  | Number of public cervical cancer awareness events conducted | 12 |
|  |  | Percentage of women undergoing period cervical cancer screening among those exposed women to occupational hazards | 50 |
|  |  | Number of providers trained in providing cervical cancer services by cadres | 2000 |
|  |  | Number of providers trained in colposcopy and LEEP per cadre | 70 |
|  |  | Number of health professional training institutions providing training modules in cervical cancer screening and preventive therapy | 12 |
|  |  | Percentage increase in number of cervical cancer screening and treatment sites | 80 |
|  |  | Increase treatment rate for precancerous lesions | 85 |
|  |  | Increase screening coverage | 72 |
|  |  | Percentage of HIV/ART clinics providing cervical cancer screening and treatment services | 90 |
|  |  | Percentage of cervical cancer screening and treatment sites without stock out of commodities used in screening and treatment services | 90 |
|  |  | Number of mentorship and supportive supervisions conducted per year | 4 |
|  |  | Number of screening/treatment clinics using visual devices for quality assurance | 60 |
|  |  | Percentage of cervical cancer screening and treatment sites whose submitted routine services data has less than 5% of inconsistencies | 95 |
|  |  | Percentage of referred women who provided feedback after receiving care | 90 |
|  |  | Percentage of facilities providing HPV based cervical | 50 |
|  |  | Percentage of women who are linked to care upon testing HPV positive | 70 |
|  |  | Percentage of women who receive diagnostic services among those referred for cervical cancer diagnosis | 90 |
|  |  | Percentage of women who receive cervical cancer treatment services among those diagnosed with cervical cancer | 90 |
|  |  | Percentage of health facilities with operational infrastructure for cervical biopsy sample collection, tissue processing and preparation for histopathologic examination among facilities providing cervical cancer control services | 90 |
|  |  | Number of lab scientists/technicians trained in tissue processing and preparation for histopathologic examination | 60 |
|  |  | Number of hospitals offering competence-based gynaecologic oncology surgical training | 4 |
|  |  | Number of gynaecologists with competence in gynaecologic surgical oncology from gynecologic oncology surgical trainings | 15 |
|  |  | Number of central hospitals with designated accomodation facilities for cancer patients and their caregivers receiving outpatient cancer treatment | 4 |
|  |  | Percentage of health facilities with tumour boards among those providing cancer treatment | 90 |
|  |  | Average time (in weeks) taken from referral to cancer diagnosis |  |
|  |  | National cervical cancer care guidelines developed, disseminated and in use |  |
|  |  | Availability of functioning supportive care programs for cervical cancer patient |  |
|  |  | Percentage of cervical cancer patients receiving supportive care among all cervical cancer patients eligible for supportive care | 90 |
|  |  | Percentage of facilities without stock out of cervical cancer treatment commodities among facilities providing cervical cancer treatment | 90 |
|  |  | Number of training programs in cancer research | 10 |
|  |  | Number of operational research studies conducted on cervical cancer | 30 |
|  |  | Cervical cancer research included in the National Research Agenda |  |
|  |  | Number of research studies on cervical cancer disseminated through symposia or research dissemination conferences | 15 |
|  |  | Cervical cancer facility reporting rate | 100 |
|  |  | Number of staff trained in monitoring, evaluation and data management per cadre | 1500 |
|  |  | Number of staff trained in cancer registration and surveillance | 30 |
|  |  | Operational national monitoring and evaluation plan for cancer registries |  |
|  |  | Linkage system between the cancer registry database and the CECAP database developed, in use and maintained |  |
|  |  | Condom use at last sexual intercourse |  |
|  |  | Number of people reached with condom use education |  |
|  |  | Number of men circumcised |  |
| Eswatini | Sexual and reproductive health annual programme report | Number of condoms distributed |  |
| Zambia | National cancer control strategic plan | Percentage of 9 – 13 year old girls completing full three-dose vaccination | Over 80% coverage of eligible girls |
|  |  | Percentage of the eligible population accessing cervical cancer service | over 80% of women of reproductive age by 2021 |
|  |  | Percentage of eligible women screened at least once with VIA | 80% |
|  |  | Percentage of VIA positive women eligible for cryotherapy completing same-day treatment. | 80% |
|  |  | Number of sites offering LEEP services | from 25 to 132/132 sites |
|  |  | Percentage of VIA positive women eligible for LEEP who complete LEEP treatment. | 95% of eligible |
|  |  | Number of sites offering VIA/Treatment | from 41 to 132/132 sites |
|  |  | Percentage of women over the age of 25years receiving mHealth messages | above 80% |
|  |  | Number eligible persons receiving cervical cancer treatment | over 80% of eligible patients |
|  |  | Number of staff capable of performing VIA plus cryotherapy | train additional 300 |
|  | Visual Inspection with Acetic Acid (VIA) and Cryotherapy: A Reference Manual for Trainers and Health Care Providers | Number of new women who received VIA screening in the target age range | 85% by 5 years |
|  |  | Percentage of new women screened with a VIA positive result positivity rate | Benchmark 5-10% a month |
|  |  | Percentage of women referred for suspect cancer | Benchmark <1%/quarter |
|  |  | Percentage of women referred for large lesions | Benchmark about 10%/month |
|  |  | percentage of eligible cc screened new women screened and treated with cryotherapy on the same day | Benchmark 80% or above |
|  |  | percentage of new VIA and cryotherapy eligible women who receive cryotherapy including SVA and those who postponed and returned) overall cryotherapy treatment rate | Benchmark 90% / month |
|  |  | Number of new women referred to another site for advanced care and treatment : overall referral rate (suspect cancer and referrals for large lesions) |  |
|  |  | Percentage of all women who have confirmed cancer after referral |  |
|  |  | Percentage of new women who received treatment for large lesions after referral |  |
|  |  | Percentage of VIA positive women who postponed cryotherapy |  |
|  |  | Percentage of VIA + women who postponed cryotherapy and returned (for those who do not return, lost to treatment follow-up, will be deduced) |  |
|  |  | Percentage of women that receive treatment that return with post-treatment complication |  |
|  |  | Percentage of previously treated women (cryotherapy and LEEP) that return for 1-year follow-up visit. |  |
|  |  | Percentage of women who return for 1 year follow up visit after treatment in previous year and now have a VIA-negative result (cure rate) |  |
| Lesotho | National multi-sectoral integrated strategic plan for the prevention and control of NCDs | HPV vaccination coverage sustained at >90% |  |
|  |  | Proportion of women screened for cervix cancer |  |
|  |  | Cancer treatment centre established in Lesotho by 2020 |  |
|  |  | Number of girls (9-13 years old) vaccinated for HPV |  |
|  | National health strategic plan | Percentage of women provided cervical cancer screening |  |
|  |  | Number of women screened |  |
|  |  | Cervical cancer screening |  |
|  | Clinical practice standards: CC prevention. CC prevention practice guidelines | Percentage of target population screened |  |
|  |  | Percentage of abnormal screening results test positivity |  |
|  |  | Percentage of facilities that are offering screening services |  |
|  |  | Percentage of health care providers trained in screening |  |
|  |  | Percentage of women with positive screening results test positivity |  |
|  |  | Treatment rate: percentage of women diagnosed with CIN2 treated |  |
|  |  | Incidence of CC |  |
|  |  | Mortality from CC |  |
|  | Guidelines for screening for cervical pre-cancer in Lesotho | Percentage of women aged 25 and above screened |  |
|  |  | Coverage: Percentage of target population screened |  |
|  |  | Smear adequacy: Percentage of all smears that are identified by the laboratory as having endo-cervical cells. |  |
|  |  | Facility coverage: Percentage of health care facilities offering screening services. |  |
|  |  | Availability of skills: Percentage of health care providers trained in screening. |  |
|  |  | Turnaround time of screening method. |  |
|  |  | Diagnosis to treatment time. |  |
|  |  | Number of women screened. |  |
|  |  | Percentage of women with positive screening results. |  |
|  |  | Screening abnormality rates: VIA/VILI positive. Atypical squamous cells (ASC-US and ASC-H), AGUS, LSIL, HSIL, and HPV abnormality. |  |
|  |  | Treatment rate: Percentage of women diagnosed with HSIL treated |  |
|  |  | Incidence of invasive cervical cancer. |  |
|  |  | Mortality rate from cervical cancer. |  |
| Zimbabwe | National cancer prevention and control strategy for Zimbabwe | HPV vaccination coverage | 85% by 2018 |
|  |  | Percentage of women 25-59 years old examined at least once for cancer of the cervix | 25% |
|  |  | Percentage of health facilities providing integrated HIV/STI/CC screening | 60% by 2016 |
|  |  | Number of staff trained in integrated CC/breast/HIV/STI service per facility | 100% provincial and district hospitals by 2016 |
|  |  |  | 100% primary health care facilities by 2018 |
|  |  | Existence of functional radiotherapy services at Mpilo and Parirenyatwa hospital | 100% functionality by 2018 |
|  |  | Availability of adequate human resources for cancer control | 70% staffing level by 2018 |
|  |  | Availability of essential affordable cancer management medicines from NatPharm |  |
|  |  | Percentage of facilities HIV/STI and cancer integrated services | 100% by 2018 |
|  |  | Percentage of clients accessing integrated HIV/STI and cancer services | 100% by 2018 |
|  |  | Number of staff trained in integrated cancer/HIV/STI early detection and management services | 100% by 2018 |
|  |  | Availability of a functional cancer database | 2015 |
|  |  | Existence of a functional referral system at all systems | 2015 |
|  |  | Incidence of CC | Down by 5% |
|  |  | Mortality of CC | Down by 5% |
|  | The Zimbabwe cervical cancer prevention and control strategy | HPV vaccination coverage for eligible girls (girls aged 11 years) | 80% |
|  |  | Percentage of districts offering vaccination | 3% |
|  |  | Screening coverage for women 30-49 years | from 13%-50% by 2020 |
|  |  | Increase in the percentage of women who have heard about CC | 79%-90% |
|  |  | Treatment rate for pre-cancer cryotherapy and LEEP | 53%-80% |
|  |  | Percentage of women eligible for LEEP or suspicious cancer who have access to histopathological diagnosis | 50% |
|  |  | Surgical treatment rate for invasive cancer | 10% of eligible women |
|  |  | Radiotherapy and chemotherapy treatment | 65% |
|  |  | VIAC outreach services |  |
|  |  | CC age-specific mortality rate | from 35.3-33/100,000 |
|  |  | CC age-specific incidence | from 56.4-52/100,000 |
|  | Guidelines for ART for the prevention and treatment of HIV in Zimbabwe | Number of people trained |  |
|  |  | Percentage of people trained still working in the content area 1 year later |  |
|  |  | Percentage of facilities offering VIA and cryotherapy |  |
|  |  | Number of district, provincial and national awareness campaigns |  |
|  |  | Number of Mass screening campaigns |  |
|  |  | Number of new women who received VIA screening in the target age range |  |
| South Africa | Cervical cancer prevention and control policy | Coverage of HPV vaccination (defined) |  |
|  |  | Incidence of oncogenic HPV infection |  |
|  |  | Proportion of primary health care facilities providing LBC |  |
|  |  | Availability of LBC services |  |
|  |  | Access to CC screening services |  |
|  |  | PHC that can provide cervical cancer screening services |  |
|  |  | Coverage of CC screening amongst eligible women |  |
|  |  | Total number of women with HG SIL |  |
|  |  | Treatment of precancerous lesions. Women with HG- SIL / CIN 2-3 who receive appropriate treatment |  |
|  |  | Proportion of women with cervical cancer still living 5 years from date of diagnosis |  |
|  |  | Incidence of cervical cancer |  |
|  |  | Mortality of CC | reduce by 20% |
|  |  | 5-year survival of women diagnosed with CC |  |
|  |  | Women with cervical cancer who receive palliative care |  |
|  | Strategic Plan for the Prevention and Control of Non-Communicable Diseases | Number of pre-sexual girls given the HPV vaccine | All age appropriate girls 100% |
|  |  | Number of women with STIs screened for CC at diagnosis and every 5 years and Number of other screened women every 10 years. | 65% of women over 30 attending public sector clinics screened. 65% of women with STIs screened soon after/at diagnosis at the 5 year intervals |
| Mozambique | National cancer control plan | Create health indicators that allow monitoring and evaluation of cancer care | |
|  | National guidelines for the prevention of cervical cancer | Screening coverage rate: | 80% |
|  |  | Number VIA positive | VIA negative |
|  |  | Number VIA positive by HIV status | Provider's ability to diagnose and recommend the correct treatment by observing images of the cervix |
| Namibia | National Multisectoral Strategic Plan for Prevention and Control of NCDs in Namibia | Coverage of vaccination against human papillomavirus (HPV) among girls aged 11 - 13 years | 95% |
|  |  | Coverage of cervical cancer screening for women between ages 30-49 years | 80%-2025 |
|  |  | Access to palliative care assessed by morphine equivalent consumption of strong opioid analgesics (excluding methadone) per death from cancer | 20% increase-2025 |
|  |  | Mortality from NCDs |  |
|  |  | Proportion of complications |  |
|  |  | Proportion of women living with HIV 30−49 years old who report being screened for cervical cancer using any of the following methods: visual inspection with acetic acid or vinegar (VIA), Pap smear or human papillomavirus (HPV) test | 70% by 2022 |
|  | National Cervical Cancer Prevention Guidelines | Numbers of women screened using VIA, Pap smear, and HPV testing |  |
|  |  | Percentage of eligible women screened |  |
|  |  | Numbers and percentages of women screened, by (five-year) age bracket |  |
|  |  | Numbers and percentages of women screened, by HIV status (positive, negative, unknown) |  |
|  |  | Number of women with abnormal screening results, disaggregated by HIV status |  |
|  |  | Number of women with abnormal screening results who receive treatment, disaggregated by HIV status |  |
|  |  | Number of health care facilities providing cervical cancer screening, and the screening methods provided |  |
|  |  | Number of health care workers trained in VIA and treatment procedures |  |
|  |  | Number of new clients with suspected cancer |  |
|  |  | Number of clients with suspected cancer referred |  |
|  |  | Number of clients with suspected cancer who were seen and evaluated |  |
|  |  | Number of clients who had confirmed cervical cancer |  |
|  |  | Number of clients treated for cervical cancer |  |
| Botswana | Botswana National Multi-sectoral Strategy for the Prevention and Control of Non-Communicable Diseases | Coverage of HPV vaccination within eligible population 11-13 | 95%-2022, 98% 2025 |
|  |  | CC screening coverage 30-49 years | 80%-2022 - 70%Pap, 30% VIA |
|  |  | Percentage screened and linked to care |  |
|  |  | Treatment of key cancers compliant with treatment guidelines | 80%-2022 |
|  |  | Opiate consumption | 30% increase-2022 |
|  |  | Number of policy changes resulting from research findings |  |
|  |  | Average referral scheduling wait times for suspected cancer |  |
|  |  | Number of stakeholders reporting on NCD indicators, % completeness and timeliness of core NCD indicators |  |
|  |  | Number of key NCD data fields integrated into existing health information systems infrastructures |  |
|  |  | Number of national registries established for all major NCDs (hypertension, diabetes, heart disease, cancer) |  |
|  |  | Incidence of CC |  |
|  |  | Percentage of CC diagnosed early | 60%-2025 |
|  |  | Access to palliative care per capita morphine consumption) |  |
|  |  | 30% increase in opiate consumption |  |
|  |  | Achievement of 80% in set targets of training NCD-relevant specialists by 2025 |  |
|  |  | Proportion of cervical and breast cancers diagnosed early |  |
|  |  | Proportion of population with NCD prevention information (awareness) |  |
|  |  | National (multi-sectoral) per capita spending on NCDs |  |
|  | Integrated health service plan | Proportion of women screened for cervical cancer |  |
|  |  | Number of cases of CC by stage |  |
|  |  | Incidence of CC |  |
|  | Five-year Comprehensive Prevention and Control Strategy.  National Cervical Cancer Prevention Programme, Botswana. | Vaccination coverage |  |
|  |  | Number and percentage of women screened among eligible women | 80% |
|  |  | Number and percentage of women with abnormal results |  |
|  |  | Number and percentage of women with abnormal results who receive treatment |  |
|  |  | Number and percentage of cervical cancer patients referred for palliative care |  |
|  |  | Number and percentage of cervical cancer patients receiving palliative care |  |
|  |  | Number and percentage of health facilities providing palliative care services |  |
