# Supplementary material for: Cervical cancer prevention in countries with the highest HIV prevalence: a review of policies
Source: BMC Public Health. 2022 Aug 10;22:1530. doi: 10.1186/s12889-022-13827-0 (PMC9367081; doi:10.1186/s12889-022-13827-0)
Supplement: Supplementary file 9 — Additional file 9. List of documents reviewed [file 12889_2022_13827_MOESM9_ESM.docx]

**Additional file 9: List of documents reviewed**

| **Country** | **Plan title** | **Year (period)** | **Source** |
| --- | --- | --- | --- |
| **Botswana** | Botswana National Multi-sectoral Strategy for the Prevention and Control of Non-Communicable Diseases | 2018-2023 | ICCP portal |
|  | Integrated health service plan: Strategy for Changing the Health Sector For Healthy Botswana | 2010-2020 | Google/Expert |
|  | Five-year Comprehensive Prevention and Control Strategy  National Cervical Cancer Prevention Programme, Botswana | 2012-2016 | Expert |
| **Eswatini** | National cancer prevention and control strategy The National Cancer Control Plan | 2019-2022 | ICCP portal |
|  | Sexual and reproductive health - Annual Program Report | 2018 | Expert |
|  | National Prevention and Control of NCDs - annual programme report | 2018 | Expert |
| **Lesotho** | National multi-sectoral integrated strategic plan for the prevention and control of NCDs | 2014-2020 | ICCP portal |
|  | National health strategic plan | 2017-2022 | Expert |
|  | Clinical practice standards: CC prevention. CC prevention practice guidelines | 2015 | Expert |
|  | Guidelines for screening for cervical pre-cancer in Lesotho | 2012 | Expert |
|  | Guidelines for screening for Cervical Pre-cancer in Lesotho | 2020 | Expert |
| **Malawi** | National Cervical Cancer control strategy | 2016-2020 | ICCP portal |
|  | Standard Operating Procedures (SOP) for CC services | developed-2019 | Expert |
|  | National service delivery guidelines for CC prevention and control | developed-2019 | Expert |
|  | Malawi National Cancer Control strategic plan | 2019-2029 | ICCP portal |
|  | Malawi Cervical Cancer Strategic plan | 2022-2026 | Expert |
| **Mozambique** | National Cancer Control plan | 2019-2029 | Expert |
|  | National guidelines for the prevention of cervical cancer | Not stated | Expert |
| **Namibia** | National Multi sectoral Strategic Plan For Prevention and Control of NCDs in Namibia | 2017/18-2021/22 | ICCP portal |
|  | National strategic framework for HIV/AIDS response in Namibia | 2017-2022 | Expert |
|  | National Cervical Cancer Prevention Guidelines, Namibia | 2018 | Expert |
|  | National Health Policy Framework | 2010-2020 | Expert |
| **South Africa** | Cervical cancer prevention and control policy | 2017 | Google |
|  | National cancer strategic framework for south Africa | 2017-2022 | Google |
|  | South Africa NCD strategic plan | 2013-2017 | ICCP portal |
| **Zambia** | National cancer control strategic plan | 2016-2021 | Expert |
|  | Zambia Consolidated guidelines for treatment and prevention of HIV | 2018 | Expert |
|  | Visual Inspection with Acetic Acid and Cryotherapy - A Reference Manual for Trainers and health Care Providers | 2015 | Expert |
| **Zimbabwe** | National cancer prevention and control strategy for Zimbabwe | 2014-2018 | ICCP portal |
|  | The Zimbabwe cervical cancer prevention and control strategy | 2016-2020 | Expert |
|  | Guidelines for ART for the prevention and treatment of HIV in Zimbabwe | 2016 | Expert |
|  | Final Addendum to the 2016 ART Guidelines | 2019 | Expert |
|  | National STI Guideline | 2019 | Expert |
