# Supplementary material for: Cervical cancer prevention in countries with the highest HIV prevalence: a review of policies
Source: BMC Public Health. 2022 Aug 10;22:1530. doi: 10.1186/s12889-022-13827-0 (PMC9367081; doi:10.1186/s12889-022-13827-0)
Supplement: Supplementary file 10 — Additional file 10. Age standardised cervical cancer incidence and mortality rates for included countries [file 12889_2022_13827_MOESM10_ESM.docx]

**Additional file 10**: Age standardised cervical cancer incidence and mortality rates for included countries

| **Country** | **Incidence rate/100,000 women-years** | **Mortality rate/100,000 women-years** |
| --- | --- | --- |
| Botswana | 34.4 | 20.1 |
| Eswatini | 84.5 | 55.7 |
| Lesotho | 56.8 | 38.7 |
| Malawi | 67.9 | 51.5 |
| Mozambique | 50.2 | 38.7 |
| Namibia | 37.4 | 22.5 |
| South Africa | 35.3 | 19.6 |
| Zambia | 65.5 | 43.4 |
| Zimbabwe | 61.7 | 43.0 |

***Source****: HPV information centre (*[*https://hpvcentre.net/datastatistics.php*](https://hpvcentre.net/datastatistics.php) *), 2021*
